# Impact of thermal stratification on airborne transmission risk of SARS-CoV-2 in various indoor environments

**DOI:** 10.1007/s12273-023-1021-5

**Published:** 2023-05-09

**Authors:** Fan Liu, Zhiwen Luo, Hua Qian

**Affiliations:** 1 School of Environment and Architecture, University of Shanghai for Science and Technology, Shanghai, China; 2 Welsh School of Architecture, Cardiff University, Cardiff, UK; 3 School of Energy and Environment, Southeast University, Nanjing, China

**Keywords:** SARS-CoV-2, temperature gradient, different-type buildings, transmission risk

## Abstract

There exist various vertical temperature gradients in different-type buildings. A holistic understanding of the impact of different temperature-stratified indoor environments on infection risk is necessary. In this work, the airborne transmission risk of SARS-CoV-2 in different thermally stratified indoor environments is assessed using our previously developed airborne infection risk model. Results show that the vertical temperature gradients in office building, hospital, classroom, etc. are within the range of −0.34 to 3.26 °C/m. In large space such as coach station, airport terminal, and sport hall, the average temperature gradient ranges within 0.13–2.38 °C/m in occupied zone (0–3 m); in ice rink with special requirements of indoor environment, the temperature gradient is higher than those in the above indoor spaces. The existence of temperature gradients causes multi-peaks of the transmission risk of SARS-CoV-2 with distancing, and our results show that in office, hospital ward and classroom, the second peak of the transmission risk is higher than 10^−3^ in most contact scenarios, while most being lower than 10^−6^ in large spaces like coach station and airport. The work is expected to provide some guidance on specific intervention policies in relation to the types of indoor environments.

**Electronic Supplementary Material:**

the Appendix is available in the online version of this article at 10.1007/s12273-023-1021-5.

## Keywords

SARS-CoV-2;
temperature gradient;
different-type buildings;
transmission risk

## 1 Introduction

The coronavirus disease 2019 (COVID-19) pandemic has raised increased awareness on the transmission of respiratory viruses in different-type indoor settings. As the pandemic continues, airborne transmission has been recognized as a main mode of the world-wide COVID-19 infections (Morawska and Cao, 2020; Greenhalgh et al. 2021). In the virus spreading via human-exhaled aerosols, as illustrated in Figure 1, especially for short-range airborne transmission, the impact of the vertical temperature distribution in indoor environments plays an important role, and has receive a growing concern in risk assessment.

The dispersion of the gas cloud exhaled by an infected patient can be described by the interaction of the respiratory emissions of source and the surroundings (Xie et al. 2007). Temperature distribution of the surrounding air affects the entertainment and stratification of the moving and dispersal of the buoyant jet flow (Bjørn and Nielsen 2002; Qian et al. 2008; Nielsen and Xu 2012). Existing studies have reported that there is a significant lock-up phenomenon of the expired airflow in indoor environments when the background air temperature is stably stratified (Gao et al. 2012; Zhou et al. 2017; Liu et al. 2020). The cloud that carries virus-laden droplets or droplet nuclei could travel a longer distance within the lock-up layer, extending the range of direct exposure of the receiver. Moreover, the lock-up layer could be closer to people's breathing zone with a stronger thermal stratification, increasing the virus concentration inhaled by receivers. Liu et al. (2020, 2021a) provided insights into the lock-up mechanism, and found that the lock-up height of the expired cloud trajectory had a clear functional dependence on temperature gradient. Fine droplets or droplet nuclei within the cloud are also trapped at a certain height, which is jointly determined by temperature gradient, initial size of droplets, and their evaporation. The lock-up phenomenon

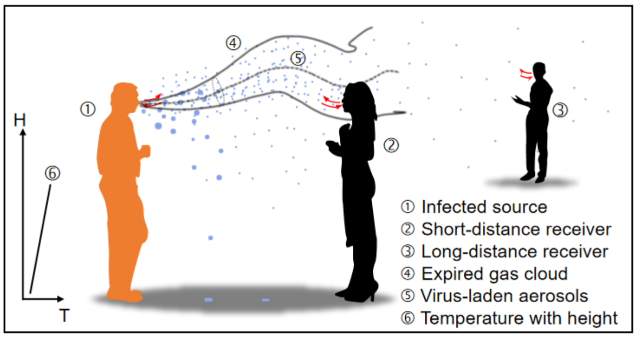

**Fig. 1** Cross infection via short- and long-distance airborne transmissions induced by vertical temperature stratification in indoor environments has ignited the re-examination of the safe threshold of physical distancing among people with close contact (Liu et al. 2021b). Therefore, the impact of vertical temperature gradient should not be neglected while assessing the transmission risk assessment of infectious diseases in thermally-stratified indoor environments.

Extensive studies on the thermal environments show that in different types of buildings, the ventilation strategy differs with the requirements for design, giving rise to various vertical temperature distributions in different indoor environments. It states in the ISO7730:2005(E) standard that the percentage dissatisfied is positively correlated with the temperature difference between human's head and ankles (ISO 2005). ASHRAE standard suggests that the temperature difference within the height of 0.1–1.8 m should also be lower than 3 °C when people are standing (ASHRAE Standard). However, numerous measurement studies suggest that in real-world scenarios, the vertical temperature gradient exists widely indoors, and is significantly related to the building types and ventilation strategies (Raftery et al. 2015; Gil-Lopez et al. 2017; Wang et al. 2019). In addition, unique functions or requirements of the indoor environment, such as indoor ice rink also make the vertical temperature varies differently (Lin et al. 2022). Furthermore, the type of the buildings also determines the activity level and respiratory rate of its occupants, occupant density and layout, positions and orientations, as well as the duration of exposure, which are all important factors in determining the distinctive characteristics of airborne transmission risk for the susceptible individuals (Ai and Melikov 2018; Nielsen and Xu 2022). However, there is still a lack of holistic understanding on the airborne transmission risk of respiratory infectious diseases in different temperature-stratified indoor environments.

In this work, we focus on the difference in the vertical temperature gradient in relation to the type of indoor environments and the resultant airborne infection risk. The short-range airborne transmission risk of SARS-CoV-2 in different thermally-stratified indoor environments is evaluated by our previously developed airborne infection risk model (Liu et al. 2021b). Instead of "just in case" lock-down closures or one-size-fits-all fixed physical distance rules, this work is aimed at providing direct comparisons of the risk level in different-type indoor environments that better reflects the multiple factors co-determining the transmission risk of COVID-19. Quantitative assessment of infection risk with physical distancing and duration of exposure is relevant to inform safe interaction in some specific occasion settings in different thermally-stratified indoor environments. It is expected to give some implications on the guidance- or policy-making to reduce suck risks during the pandemic.

## 2 Material and methods

### 2.1 Indoor vertical temperature profiles in different-type of buildings

We firstly collect the indoor air temperature data from the published literature based on the measurements in various settings or ventilation designs with two aims: (1) to show the wide existence of the temperature stratification in the real-world indoor environments and its necessity in assessing virus transmission rather than simply assume the indoor air to be well-mixed; and (2) to provide input data for the prediction model based on the on-site measurement data reviewed. The data collection is conducted as an investigation of relevant publications in peer-reviewed journals and conference proceedings. All the papers published after 2000 are initially screened and evaluated based on the title and abstract. There are no strict geographical restrictions on the field measurements of indoor temperature in the preliminary screening stage. Particular attention is paid to the studies that complied with the following criteria: well-described building/indoor environment information, well-justified research methodology, and quantitatively-presented vertical temperature profiles. The following studies are excluded: (1) not field measurement of the indoor air temperature distributions (e.g., reviews, theoretical predictions and CFD simulations); (2) with no quantitative vertical temperature data; and (3) duplicated studies. With the aid of a data extracting software "GetData Graph Digitizer 2.26" (Digitizer 2020), temperature points are retrieved from the original curves for comparisons and further analysis.

### 2.2 Mathematical models for inhalation risk prediction

An infection-risk-based mathematical model proposed in our previous study (Liu et al. 2021b) is employed in this work to examine the dynamics of the transmission process of SARS-CoV-2 in thermally stratified indoor environments. Whereas the details of the model can be found therein, a brief description for the key stages of disease transmission included in the model is provided here: First, the exhaled gas cloud ④ from the infected source ① is simplified as a buoyant jet flow (Figure 1). The velocity and temperature of the jet flow and its carried pollutant concentration on the trajectory (indicated by point ***c*** in Figure 2) are solved by an integral model formulated by a group of ordinary differential equations (ODEs). The distribution of the temperature, velocity and concentration at a certain point in the ambient environment (indicated by point ***p*** in Figure 2) can be obtained by Gaussian assumption on the cross-sectional plane. Second, the transport and evaporation of virus-laden droplets and aerosols ⑤ during the transmission process with the jet flow are expressed as another set of coupled ODEs with mass and heat transfer equations between the droplet surface and the combined background of exhaled cloud and ambient air. Third, the inhalations of the receivers ② are determined by positions and relative orientations between ① and ②, respiratory rate and exposure time of ②. Fine droplets and droplet nuclei in the air passages of the receiver's respiratory system deposit by various mechanisms that are size-dependent in the empirical formula. Finally, the deposition of infectious viruses in the respiratory tract results in infection with a dose-response relation $p = 1 - \exp(-\sigma\mu)$, where the factor $\sigma$ is the infectivity factor which is the inverse of the number of viruses that can initiate an infection, and $\mu$ is the exposure dose. As a result, the infection of the receiver can be obtained which varies with distancing to the source.

To consider the impact of the vertical temperature gradient of indoor air, an integral method is used in the first stage of the risk assessment model for the conservation of mass, momentum, buoyancy and scalar quantities in the exhaled buoyant jet flow, in which the impact of the air temperature stratification is embodied in the conservation principle for the jet flow in the form of vertical density gradient. The empirical formulas for jet flow are frequently used for the travelling trajectory of expiratory airflow in many studies (Xie et al. 2007; Chen et al. 2020), but wherein the indoor air is assumed to be well mixed without vertical temperature gradient. Here, we assume the air density as a function of the temperature, so the vertical density gradient can be obtained by the vertical temperature profiles. To highlight the impact of the vertical temperature gradients in indoor environments, the transmission risk of SARS-CoV-2 without considering the specific temperature profiles (i.e., assuming the indoor air temperature is uniformly distributed) is also calculated for comparison.

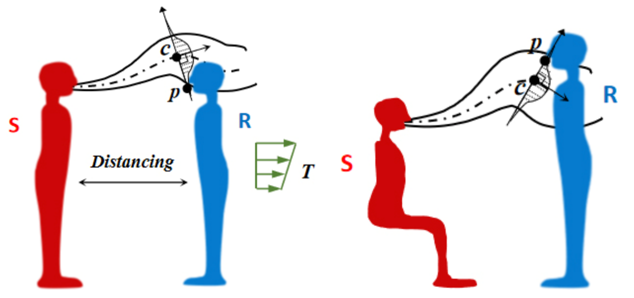

**Fig. 2** Gaussian assumption for the parameters on the cross-sectional plane of exhaled buoyant jet flow: S represents infected source and R represents susceptible receiver; the distancing refers to the distance between two human mouths

## 2.3 Variables and outcome analysis

Models for risk predictions of infectious diseases have played a vital role in developing public health strategies for disease control and prevention (Grassly and Fraser 2008; Buchwald et al. 2020; Zhao et al. 2022). However, the parametric uncertainty affects the robustness of a modelling study's qualitative conclusions (Wu et al. 2013). In this work, considering the variations of the temperature profiles measured in the real-world buildings in existing studies, and the variability of other inputs of the risk prediction model (e.g., viral load of the infected source, and duration under each exposure scenario), Monte Carlo (MC) method is applied to test the robustness of the findings by elucidating the plausible range of the transmission risk in different types of indoor environments, for forecasting purposes, with the flowchart shown in Figure 3.

First, the probability density functions (PDFs) characteristics of each parameter in the airborne infection risk model are given as the inputs for the MC simulations, such as the number and size distributions of droplets in different exhalation modes, viral load of SARS-CoV-2, exposure time

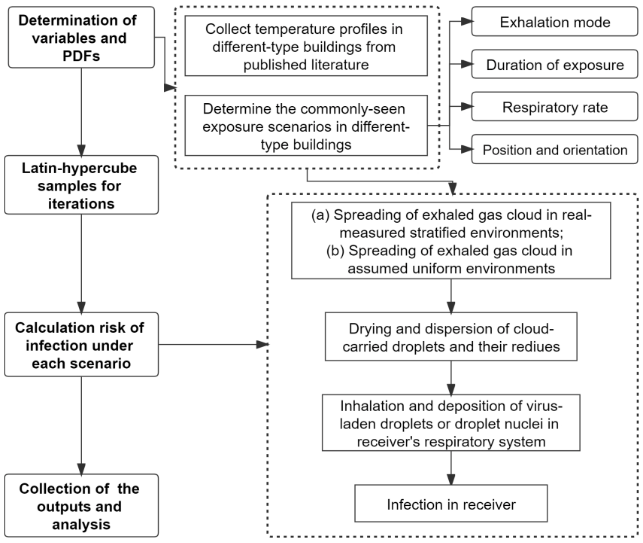

**Fig. 3** Flowchart of the MC simulations for risk assessment of the receiver, and temperature gradient of the indoor air. Li et al. (2020a) suggested that 79% of the actual identified cases were infected by individuals with “mild, limited, or no symptoms”. Oran and Topol (2020) also stated that asymptomatic persons seem to account for approx. 40%–45% of SARS-CoV-2 infections. The abundance of speaking-generated droplets, combined with its high viral load in pre- and asymptomatic individuals strongly implicates the airborne transmission of SARS-CoV-2 via speaking as the primary contributor to its rapid spread (Stadnytskyi et al. 2020; Bax et al. 2021). Therefore, in this work, the infected source is assumed asymptomatic in all calculated scenarios, and the number and size distributions of exhaled droplets refer to the existing data measured under human speaking. The available data of the viral load of SASR-CoV-2 in respiratory secretions of an asymptomatic patient shows a wide range of variation and here is specified as probabilistic with normal distributions. Duration of exposure of the receiver depends on the contact scenario with the infected source, and we assume it to be uniformly distributed. Given this, several commonly-seen contact scenarios between two persons are given in each building type for the risk prediction. Considering that there is limited statistical data of the exposure time of people in real-world exposure scenarios in different-type indoor environments, the upper and lower limits of the uniform distribution of the exposure time for the risk prediction is taken based on field observations, video data or and surveys from the occupants. To obtain the PDF of the indoor temperature gradient in different-type buildings, the measured discrete temperature data with height in each screened reference is firstly retrieved. We assume that the air temperature linearly distributes between two adjacent measuring points in vertical direction, so the temperature gradient is calculated using the temperature measured at two adjacent heights, i.e.:

$$\mathrm{grad}T = \frac{T_1 - T_2}{h_1 - h_2}$$

where grad$T$ the temperature gradient (°C/m) between the measuring height $h_1$ and $h_2$ (m) in the reference, $T_1$ and $T_2$ (°C) are the air temperature at $h_1$ and $h_2$ (m), respectively. A range of the *grad T* for each type of indoor environment can therefore be obtained for the following MC simulations of the infection risk. The key variables and values for risk calculation are given in Table 1. More details on the modeling parameters can be found in the reference (Liu et al. 2021b).

Second, sampling is conducted from the above PDFs for each variable for performing MC simulations. Assuming the above input parameters are independent from each other. To improve sampling efficiency and monitor sampling convergence, the infection risk statistics are firstly simulated with different Latin hypercube samples (LHS) and finally a total of 15,000 LHS from the variable distributions are generated under each simulation, and the parameter variability are propagated through MC simulations of the airborne infection risk model under all given contact scenarios. Finally, the simulated risk is collected and given in the form of a guide picture of airborne transmission risk to offer the basis practical for making specific policies in relation to the types of indoor environments.

## 3 Results and discussion

### 3.1 Indoor vertical temperature profile varies with building type, function and ventilation system

From 3,178 papers identified in the search, we reviewed 183 full texts involving field measurements of the air temperature in indoor environments of different-type buildings. From these we included 30 studies directly reporting the vertical temperature profiles in occupied zones (0–3 m) of 11 types of indoor environments, including office, hospital, classroom, lab. building, factory hall, coach station, airport, shopping mall, sport hall, ice rink and swimming pool. The comparison of the retrieved temperature data in these studies on the 11 types of indoor environments is shown in Figure S1 in Appendix S1. Significant temperature variations over heights can be found across many types of indoor environments within the same type of indoor space. The

**Table 1** Exposure scenarios between the source and receiver in different-type buildings

| Parameter | Values | Distribution | Reference(s) |
|---|---|---|---|
| Viral load of SARS-CoV-2, $C_{\mathrm{RNA}}$ (copies/mL) | $10^7$, 25% | Normal | Pan et al. 2020; To et al. 2020; Biguenet et al. 2021 |
| Exhalation velocity, $u_0$ (m/s) | Breathing: 1.0<br>Speaking: 3.9 | Constant | Chao et al. 2009; Archer et al. 2022 |
| Volumetric breathing rate, $q_{\mathrm{ex}}$ (m$^3$/h) | Seated (rest): 0.49<br>Standing: 0.54 | Constant | Adams 1993 |
| Exposure time, $t_d$(s) | See Table 2 | Uniform | — |
| Infectivity factor of SARS-CoV-2, $\sigma$ | 0.01–0.1 | Uniform | Buonanno et al. 2020a |

vertical temperature difference is attributed to the indoor air distributions induced by different ventilation systems, airflow patterns, internal thermal load produced by equipment, number of diffusers, time of day, etc.

In indoor space with a low height (< 3 m), it is widely accepted that indoor air was well mixed with mixing ventilation (MV), in which case the air temperature is distributed uniformly (Qian et al. 2006; Bjørn and Nielsen 2002). In the simulations of Lu et al. (2020), the temperature showed little variations with height in a two-bed hospital ward with MV, however, their measured temperature exhibited an obvious increase from 23 °C to 25.5 °C in the breathing zone (1.3–1.7 m above the floor in their work) under the same conditions. In some indoor environments with natural ventilation (NV), DV or under-floor air distribution (UFAD), the low-momentum air supply is attached to the floor, and a vertical temperature stratification is established with warmer air rising and cooler air descending due to thermal buoyancy. As the results from Raftery et al. (2015) show that a temperature difference of up to 13 °C was observed in a 3-m-high office room with DV or UFAD, see Figure S1(a) in Appendix S1. A classroom ventilated with displacement natural ventilation (DNV) system had a vertical temperature difference of approx. 3 °C within 0.1–1.1 m at the location of students as studied by Wang et al. (2014), see Figure S1(c) in Appendix S1.

In some large space with a high room height (>8 m), upper-side nozzle air supply (UNAS) system, sidewall nozzle air supply (SNAS) system, stratified air distribution system (STRAD) and JV are frequently used for a high speed of the supply air or for energy conservation purpose. The difference of the vertical temperature profiles may be caused by the various airflow patterns, heat sources, with/without occupants and measurement time. For example, Tong et al. (2020) found a small temperature difference at 7:00 a.m., but a large temperature difference of 4 °C at 11:00 between the heights of 0.5–8.5 m in the atrium of a coach station. Zhao et al. (2020) and Gil-Lopez et al. (2017) studied the thermal environments in airport terminals, and found that in addition to the impact of ventilation system, the air temperature distributions had an obvious difference in different function areas, as shown in Figure S1(e) in Appendix S1. Zhao et al. (2020) also found that the vertical temperature difference from 0 to 3 m was of about 6.5, 5.7 and 1.5 °C in three arrival halls, respectively, while only a small temperature difference of 0.2 °C in departure hall. Indoor ice arena is another typical type of large space with special requirements of indoor environments, and has gradually become a hot topic with the development of winter sports around the world. To prevent the ice surface from melting or frosting (ASHRAE 2009), the air near the ice surface needs to be dry and cold. Palmowska and Lipska (2016) and Sormunen et al. (2007) proposed that the general temperature and relative humidity are usually 10%–12 °C and 40%–65% respectively in ice arenas. As our collected data compared in Figure S1(f), the air temperature in sportsman's breathing zone is only approx. 5–15 °C, much lower than the 19–23 °C in other-type indoor environments.

These findings suggest that in real-world indoor space, the vertical temperature distributes non-uniformly in most cases. A greater vertical temperature difference than the acceptable level in the occupied zone not only brings the thermal discomfort, also may not be conducive to the diffusion and dilution of pollutants. In this work, we focused on the short-range airborne transmission of SARS-CoV-2 among people, so special attention was paid to the temperature gradients in the occupied zone (0–3 m). The temperature gradients calculated using the method in Section 2.3 "Variables and outcome analysis" are summarized in Figure 4. It shows that in the indoor space within 3-m-height, such as office room, hospital wards or consultation

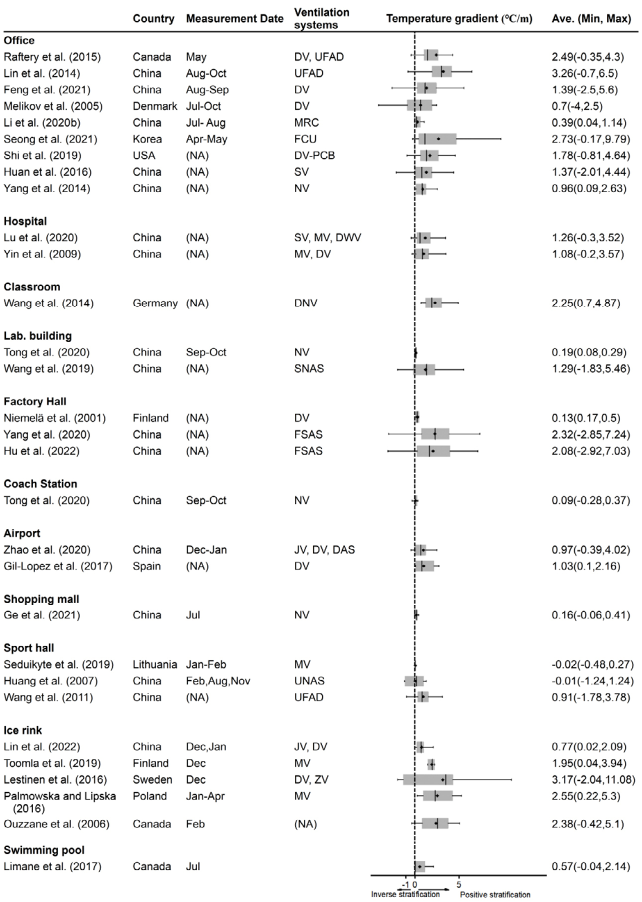

**Fig. 4** Thermal stratification intensity in occupied zones (0–3 m) of different-type indoor environments. SV = stratum ventilation, MRC = modular radiant cooling system, FCU = fan coil units, PCB = passive-chilled-beam, DWV = downward ventilation, FSAS = floor-level sidewall air-supply system, and ZV = zoning ventilation rooms and classroom, the temperature gradient has a wide range with the averaged values of 0.39–3.26 °C/m in different studies. By comparison, in large space higher than 8 m, such as laboratory building, coach station, airport, shopping mall and sport hall, the temperature gradient in occupied zone is in a range of 0.13–2.38 °C/m, smaller than that in the indoor space less than 3 m. However, as the above temperature profiles show, the temperature difference is larger in these types of indoor environments, which implies that the temperature stratification in unoccupied zones (>3m) is more obvious than that in occupied zones in large ventilated space. It's worth noting that in indoor ice rink, the vertical temperature gradient is larger than those in other types of large space, ranging from 0.77 °C/m to 3.17 °C/m. The results suggest that in indoor space where people occupy for a long time the temperature gradient exits widely and varies significantly with the type of indoor environments.

## 3.2 Transmission risk of SASR-CoV-2 in different thermally stratified indoor environments

In the MC simulations, to reduce the number of uncertain parameters in the theoretical prediction model, the types of indoor space with relatively fixed layouts, respiratory modes of occupants, and their positions and orientations are targeted for risk predictions so that the findings have general applicability. The importance of the relative exposure for individual inhalation risk to the expiratory pollutant in thermally-stratified indoor environments have been addressed by many experimental and theoretical studies (Bjørn and Nielsen 2002; Nielsen and Xu 2012; Liu et al. 2019). Here, commonly-seen exposure scenarios and duration of occupants in different types of indoor environments are summarized in Table 2. The respiratory activities are 1 m/s and 3.9 m/s when the infected source is breathing and speaking, respectively (Xie et al. 2007; Chao et al. 2009). The temperature of the exhaled air is assumed to be 35.1 °C (Popov et al. 2007). The size and number distributions of the exhaled aerosols are referred to the classical experimental dataset by Duguid (1946). Close face-to-face interaction is a typical high-exposure scenario, so we assumed that the receiver is in the direction of the buoyant jet flow exhaled from the infected source in all calculation scenarios, and both are not wearing face masks.

### 3.2.1 *Office environment*

Figure 5 describes the spread probability of SARS-CoV-2 resulting from close contact with a pre-symptomatic infected

**Table 2** Exposure scenarios between the source and receiver in different-type buildings

| Building type | | Relative postures (Source to receiver) | Source | Receiver | Exhalation mode | Duration of exposure |
|---|---|---|---|---|---|---|
| Office | Meeting room | Seated [1] to seated | Attendee | Attendee | Speaking | 0.5–1 h/1–2 h [3] |
| | | Seated to standing [2] | | | | 0.5–1 h/1–2 h |
| Hospital | Consultation room | Seated to seated | Patient | Doctor | Speaking | 3–5 min |
| | Dentist's office | Reclining [4] to standing | Patient | Doctor | Breathing | 0.5–1 h |
| | Ward | Seated to seated | Patient | Visitor | Speaking | 5–15 min |
| | | Seated to standing | | Doctor/HCW [6] | Speaking | |
| | | Lying [5] to Lying | | Patient | Breathing | 4–6 h |
| Classroom | In class | Seated to standing | Student | Teacher | Breathing | 45 min |
| | Break time | Seated side by side | Student | Student | Speaking | 5–10 min |
| Coach station | Waiting hall | Seated to seated | Passenger | Passenger | Breathing | 20–30 min |
| | | Seated to standing | | | Breathing | |
| | | Standing to standing | | | Breathing | |
| Airport | Departure hall | Seated to seated | Passenger | Passenger | Breathing | 1–2 h |
| | | Seated to standing | | | Breathing | |
| | | Standing to standing | | | Breathing | |
| | Check–in hall | Standing to standing | | Worker | Breathing | 1–5 min |
| Ice rink | Competition field | Seated to seated | Player | Player | Speaking [7] | 15 min |
| | | Seated to standing | Player | Player/Coach | Speaking [7] | |

[1] The exhalation height of a seated source is 1.18 m. [2] The exhalation height of a standing source is 1.75m. [3] Short and long duration of exposure in meeting room. [4] The exhalation height of a reclining source is 1 m. [5] The exhalation height of a lying source is 0.8 m. [6] HCW = health care worker. [7] Only the exposure scenario of motionless players during the half-time intervals is calculated in ice rink, and speaking here signifies a more violent exhalation of players than breathing.

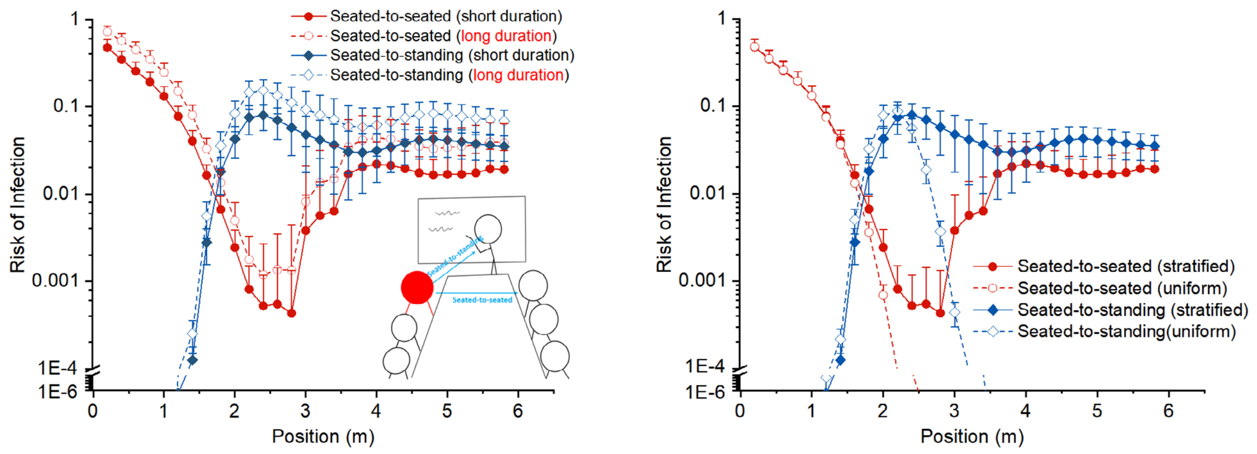

(a) (b)

**Fig. 5** Cross-infection risk in office environment (error bars represent one standard deviation from the mean): (a) with different duration of exposure in thermally stratified environment; (b) comparison of risk in real-measured stratified environment and assumed uniform environment person (seated) during a meeting in office environment. The MC simulations were performed for the two scenarios, i.e., seated source faces to seated receiver and seated source faces to standing receiver during a meeting lasting 0.5–1 h (short duration) and 1–2 h (long duration). The mean infection risk is the averaged value of the MC simulation results at different distances. The error bars in Figure 5 and the following Figures 5–8 are attributed to the uncertainty of variables in the risk prediction model, as presented in Table 1. Some studies on contact tracing, for example, consider thresholds of 5–15 minutes beyond which the infection risk increases (Cheng et al. 2020; Doung-Ngern et al. 2020). Here, we gave more quantitative details on the impacts of exposure time, as the results shown in Figure 5(a). Cross-infection risk of susceptible people increases obviously with the exposure time, and in the present settings, the transmission risk of the receiver after a long duration is about twice as high as that after a short duration. For example, at a physical separation of 1 m between the infected and the exposed individuals that are sitting on opposite sides of the table, the cross-infection risk increases from around 0.13 to 0.24 as the duration prolongs to 1–2 h. In addition, Figure 5(a) also shows that the relative posture of source and receiver is another key factor affecting the risk level. Simulated results show when the infected source is talking to a receiver who is seated across the table or seated side by side, the cross-infection risk induced by the exhaled virus from the source is mostly larger than $10^{-3}$. In detail, the averaged risk of the receiver seated across the table is up to 0.13 (assuming the width of the conference table is 1 m), while a higher risk of approx. 0.3 exists for a receiver sitting by the source when they are in discussion. However, for the standing speaker during the meeting, the cross-risk induced by a seated source peaks at a distance of 2–2.4 m, with the value of 0.04–0.08, while it becomes lower over shorter distance. It implies that the size of the conference table, layouts of meeting room and office space, especially in open office environments are vital for transmission risk of respiratory diseases, which should be paid special attentions in the early designs in response to sudden outbreaks of epidemic.

We compared the risk levels in real-world thermally-stratified indoor environments and in assumed thermally uniform environments, as results shown in Figure 5(b). It indicates that for seated receivers who are in close proximity with a seated source, it seems that the impact of the temperature gradient on the cross-infection risk is magnified as the physical separation increases to > 2 m, i.e., there is a second-high level rather than continuously decreasing at about 4 m. For standing receivers, the infection risk peaks at a distance of 2.2 or 4.8 m with the source, that is, the risk shows multi-peaks with the distancing, however, the assumption that the temperature is distributed uniformly could underestimate the short-range distance of SARS-CoV-2. It implies that in the real world, vertical temperature gradient is essential for risk assessment, which makes the risk vary more complexly than that in an ideal uniform environment. The above results also support the hypothesis that asymptomatic individuals who do not cough or sneeze can spread the infection (Rothe et al. 2020; Li et al. 2020a). In office buildings with fixed layouts, size of tables and arrangement space may promote the short-range airborne transmission.

### 3.2.2 *Hospital environment*

The outbreak of COVID-19 has created huge strain on HCWs treating infected patients. The protection of HCWs in hospitals from infection is strategic for the management of the pandemic. We simulated the cross-infection risk in consultation room, dentist's office, and hospital ward, as the results shown in Figure 6. It can be seen that in consultation room where the doctor and patient sit face to face, and talk to each other, the risk of infection is larger than 0.01 within 1 m, even with a few minutes (3–5 min) of exposure. However, in another close contact scenario in dentist's office where the doctor is exposed to the exhaled virus from a reclining and breathing patient for longer time (0.5–1 h), the risk level is instead lower than $10^{-5}$. This is mainly because emissions with large force can generate a high momentum to move the virus-laden droplets faster and further in air, and release more droplets at the same time (Archer et al. 2022). The exposure of the receiver to higher droplet concentrations (or equivalently higher viral loads) for normal speaking hints at the role of asymptomatic patients in infection transmission. Similar results can also be found in hospital ward. During visiting time or ward rounds, the risk of receivers caused by a speaking source is significantly higher than that by a source who is bed-lying in break for long time. It is interesting to note that maintaining a physical distance of <2 m for standing HCWs during ward rounds may reduce the probability of infection. It also implies that the evidence base for current guidelines is sparse, and our simulations do not support the 1–2-meter (≈3–6 feet) rule of spatial separation in some real-world scenarios, other physical interventions such as wearing face masks may be more effective in these cases.

### 3.2.3 *Classroom environment*

Figure 7 shows the spread risk of SARS-CoV-2 from an infected student to a standing teacher in class and to a seated-side-by-side desk mate during break time. It agrees well with the above findings that the cross-infection risk induced by short duration of "violent respiratory event" (talking to each other between two students seated-to-seated in break time with the transmission risk of $>10^{-3}$) could be higher than that by long duration of "mild, limited, or no symptoms" (breathing student in class with the transmission risk of $<10^{-6}$). At this point, it suggests that the impact of the emission force on risk level is more obvious than the duration of exposure. This finding addresses the important role of face coverings in these occasions in mitigating the transmission risk by trapping the virus-laden droplets and reducing the outward emissions (Ueki et al. 2020; Liu and Qian 2022).

### 3.2.4 *Coach station and airport environment*

Figures 8(a) and (b) show the results in coach station and airport, respectively, where the exposure scenarios of passengers are similar. In waiting hall or departure hall, most passengers remain at rest with limited virus released, and the resulted spreading risk is generally lower than $10^{-6}$, even in the check-in hall when the workers are in close proximity to the passengers. The results along with the above findings from Figures 5–7 can help explain why indoor case clusters have been mostly reported in churches (Miller et al. 2021), restaurants (Li et al. 2021), auditorium (Huang et al. 2022) and dormitory (Chew et al. 2020) where the indoor air distribution is more easily affected by ventilations and people might sing, laughing or speak loudly. By comparisons, it is interesting to noted that few reports of outbreaks were reported in classrooms, at station halls, on train carriages or aircraft cabins, which may reflect the relatively low risk because speaking or more violent exhalations are limited in such types of indoor places.

### 3.2.5 *Indoor ice rink*

Indoor ice rink shares some characteristics with the above building types, while it is distinctive in a number of ways, including the ventilation strategies, microclimate conditions

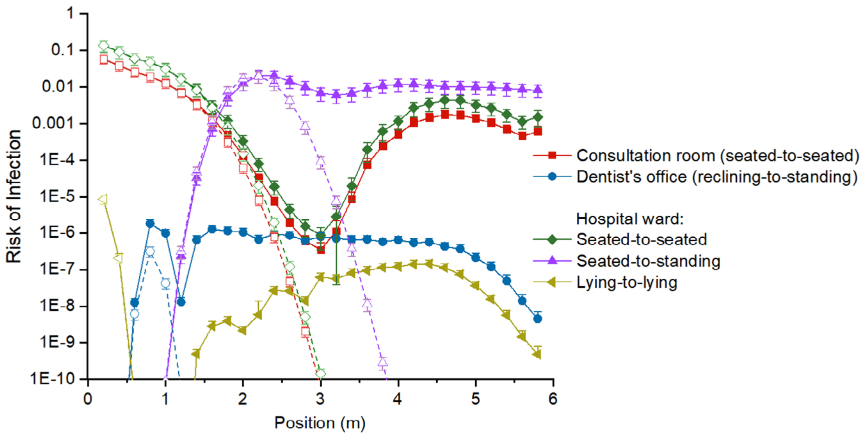

**Fig. 6** Cross-infection risk in hospital environment. Points not shown in the figure indicates the averages are less than $10^{-10}$. Dotted lines show the results in an ideal uniform indoor environment

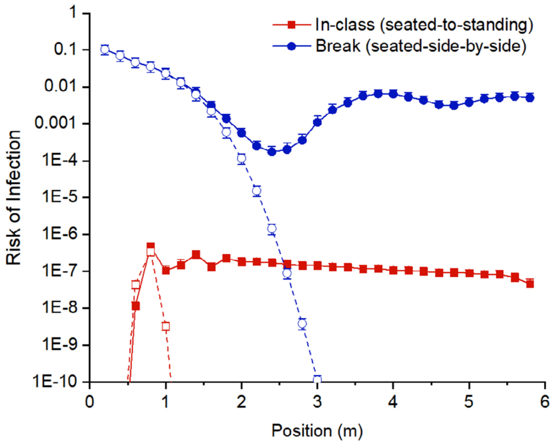

**Fig. 7** Cross-infection risk in classroom environment. Dotted lines show the results in an ideal uniform indoor environment and usage patterns of the people inside. Cold and dry air near the ice surface in competition field causes complex phenomena of air movement and pollutant dispersion. In this work, the infection risk among the ice hockey players is evaluated by considering the impact of the vertical temperature gradient in ice rinks, as results shown in Figure 9. It shows that when players have a break on the bench in halftime, the heavy emission of virus and inhalation rate can induce a high-risk level of >0.1 to another seated player at a close distance (<0.5 m). The strong thermal stratification causes multi-peaks of the risk with the distancing under the seated-to-standing exposure mode, i.e., the risk reaches second- and third-high levels at about 3.5 and 5 m, respectively. The peak of the transmission risk in the ice rink remains a high level of $>10^{-4}$, slightly lower than the risk levels in office building due to short duration of exposure, but much higher than those in coach station and airport terminals. The multi-peaks of the risk is induced by the lock-up phenomenon of the exhaled virus-laden aerosols in the thermally-stratified environment. Similar locking phenomenon of pollutants in ice rink was also observed in previous studies, for example, Toomla et al. (2019) visualized a stagnation phenomenon of air by operating a smoke generator on the ice surface. After about two minutes, the smoke settled at about 10 feet above the ice surface. Yang et al. (2000) numerically simulated the air velocity and temperature distributions above the ice surface, and also highlighted the accumulation of air pollutants in the breathing zone of the players, which potentially increases the pollutant inhalation of players.

It should be noted that the cross-infection risk among players in game time was not assessed in present work because the dynamic characteristics of the airflow and the number and size distribution of droplets exhaled by a moving source has been not been available yet. However, it can be inferred from the present results that ice hockey involves vigorous physical exertion accompanied by deep, violent exhalations with higher momentum during the game than those in halftime, which implies higher emission and inhalation of the (virus-laden) aerosols for moving players. An Italian study estimating the rate of SARS-CoV-2 emission by infected persons based on viral load in the mouth showed that during heavy exercise, a high viral emission rate can be reached during oral breathing (Buonanno et al. 2020b). A case report from an outbreak of COVID-19 associated with a recreational ice hockey game in the Tampa Bay, Florida described during the 5 days after the game, 15 persons (14 of the 22 players and a rink staff member) experienced signs and symptoms compatible with COVID-19 (Atrubin et al. 2020). Although there are limitations on investigations of this outbreak case, our findings together with the earlier reports show that the intensive temperature stratification of the indoor air, vigorous exhalations and virus emissions of the source, and

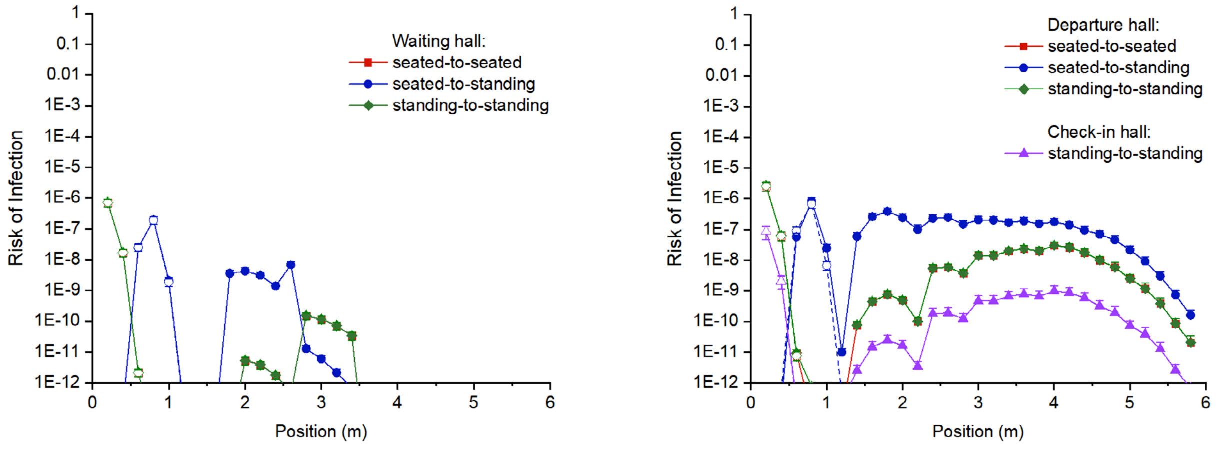

(a) (b)

**Fig. 8** Cross-infection risk in: (a) coach station; (b) airport. Dotted lines show the results in an ideal uniform indoor environment

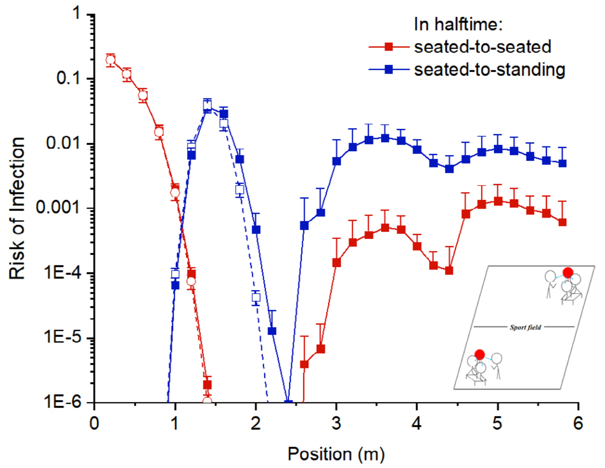

**Fig. 9** Cross-infection risk in halftime during ice hockey game. Points not shown in the figure indicates the averages are less than $10^{-6}$. Dotted lines show the results in an ideal uniform indoor environment.

close contact between players in ice rink may pose an increased risk during the COVID-19.

## 3.3 Implications for COVID-19

The risk prediction model we proposed includes the major stages of respiratory infectious disease transmission via short-range route, therefore, it is applicable to different airborne diseases carried by expiratory aerosols with available virus dynamics and infection rate, such as IAV, SARS-CoV-1 and HCoV-229E. Many viral kinetics of SARS-CoV-2 are now emerging and available (Pan et al. 2020; Biguenet et al. 2021; To et al. 2020), and we targeted the transmission risk of SARS-CoV-2 that contributes the going-on COVID-19 pandemic to provide some useful evidence-based guidelines for making policies or strategies. In response to the outbreak of COVID-19, widespread closures occurred in public space including schools, stations, airports and restaurants. However, as we have shown, the one-size-fits-all closures are costly and severely damaging to the economic prospects across industries and countries (Onyeaka et al. 2021). In this work, we compared the transmission risk of SARS-CoV-2 in relation to the building types to make the prevention policy more specific and targeted to reduce social and economic cost. We found that the existence of thermal stratification in different types of indoor environments has important impacts on the transmission risk with more guiding significance in real-world scenarios.

Table 3 presents a guide to the transmission risk level in different types of buildings, with the specified values for each color attached in Table S1 of Appendix S2. In this work, the short-range airborne transmission of SARS-CoV-2 was targeted, therefore, the risk values within a proposed 6-foot (2 m) separation (Qureshi et al. 2020) for controlling the close contact transmission during the COVID-19 pandemic were targeted, and the assessed risks at distance of >2 m were also given for comparisons. As seen in Table 3, the relative distancing between the receiver and infector is divided into five intervals, i.e., 0–0.5 m, 0.5–1 m, 1–1.5 m, 1.5–2 m and >2 m, to clarify the risk variation when the receiver is in close proximity to the source. The risk value for each box is obtained by averaging the MC simulation results at discrete distances within the intervals. The choice of an acceptable infection risk for SARS-CoV-2 is difficult and certainly questionable. Here, considering the mortality rate of SARS-CoV-2, the estimated risk of lower than $10^{-6}$ is supposed to be acceptable (Buonanno et al. 2020a), which is an order of magnitude lower than the guideline values for genotoxic carcinogens on the upper bound estimate of an excess lifetime cancer risk of $10^{-5}$ (WHO 2011), i.e., the "medium" value in Table 3 is $10^{-6}$, "high" values mean an infection risk of larger than $10^{-6}$ and "low" means an infection risk lower than $10^{-6}$. These estimates apply to the situations when everyone is asymptomatic, assuming the infected people have been identified and (self-) isolated. The result shows that the risk level varies with the type of indoor environments. In office building, hospital ward and classroom, the transmission risk is higher than that in large space such as coach station and airport. However, in sport space, both the source and receiver in motion with violent exhalations and high respiratory rate, together with a deep vertical temperature stratification such as in indoor ice rink, the infection risk of the receiver is higher than those in other-type large space. Jones et al. (2020) provided a guide how the risk levels of SARS-CoV-2 transmission from asymptomatic people may vary with setting, occupancy level, contact time, and whether face coverings are worn, based on which more quantitative results are shown using a risk-based prediction model in our work.

The risk comparison of different-type building environments also implies that in public occasions the risk level relies heavily on individual behaviors. In situations with high risk (indoor air with intensive vertical temperature stratification, noisy surroundings and more frequent violent respiratory exhalations, long contact time, relative postures with breathing heights and no facing coverings), physical distancing may not always be effective; especially in crowded indoor space such as office, hospital wards and restaurants, the short-range transmission route with high risk may opportunistically evolve into long-distance airborne transmission or even cluster transmission by poor ventilation indoors (Li et al. 2021). Consistent conclusion was given by Jones et al. (2020) that in the highest risk situations with poor ventilation, high levels of occupancy, prolonged contact

**Table 3** Cross-infection risk of SARS-CoV-2 from asymptomatic people in different types of indoor environments

| Building type | | Exposure scenario | Physical separation | | | | |
|---|---|---|---|---|---|---|---|
| | | | 0–0.5 m | 0.5–1 m | 1–1.5 m | 1.5–2 m | > 2 m |
| Office, short duration of exposure | Meeting room | Seated to seated | | | | | |
| | | Seated to standing | | | | | |
| Office, long duration of exposure | Meeting room | Seated to seated | | | | | |
| | | Seated to standing | | | | | |
| Hospital | Consultation room | Seated to seated | | | | | |
| | Dentist's office | Reclining to standing | | | | | |
| | Ward | Seated to seated | | | | | |
| | | Seated to standing | | | | | |
| | | Lying to Lying | | | | | |
| Classroom | In class | Seated to standing | | | | | |
| | Break time | Seated side by side | | | | | |
| Coach station | Waiting hall | Seated to seated | | | | | |
| | | Seated to standing | | | | | |
| | | Standing to standing | | | | | |
| Airport | Departure hall | Seated to seated | | | | | |
| | | Seated to standing | | | | | |
| | | Standing to standing | | | | | |
| | Check-in hall | Standing to standing | | | | | |
| Ice rink | Competition field | Seated to seated | | | | | |
| | | Seated to standing | | | | | |

**Cross-infection risk level**

Low Medium High time, and no face coverings, physical distancing beyond 2 m and minimizing occupancy time should be considered. In addition, the importance of indoor ventilation becomes obvious. Buildings with potential high transmission risk among its occupants may be equipped with better ventilation systems (with pandemic time operation mode) and filtration to reduce the opportunities of long-distance airborne transmission and super spreading events. Moreover, other physical interventions, such as setting partitions to cut off the transmission route and staggered seats to reduce the inhalation dose are more recommended than solely increasing the separation distance. By comparison, in some large public space where silence is required or the occupants remain at rest with mild respiratory modes, e.g., in waiting hall of station and airport, the low risk level suggests that less stringent distancing is likely to be adequate. People with mild exhalations have low viral load, with the short-distance airborne transmission yielding little to no risk of onward transmission.

## 4 Conclusions and limitations

This is probably the first study in which the thermal stratification of indoor air in real-world indoor environments of different-type buildings has been considered in the risk prediction of SARS-CoV-2. Different from the one-size-fits-all 2-m physical distancing rule derived from simple calculations by assuming a uniform distribution of indoor air, we highlighted the difference in the transmission risk levels of SARS-CoV-2 with the social distancing in specified exposure scenarios in various thermally -stratified indoor environments. It is concluded that: (1) the vertical temperature gradients widely exist in indoor environments, and vary significantly with the types of buildings, especially in occupied zones (0–3 m); (2) in the indoor space with a height of <3 m, the vertical temperature gradient in occupied zone shows a larger variation range of 0.39–3.26 °C/m, while in large space of >8 m, the temperature stratification in unoccupied zones (>3 m) is more obvious than that in occupied zones; (3) the consideration of temperature gradient in the prediction model makes the risk shows a multi-peak tend rather than monotonic decreasing with the distancing to source; (4) in office, hospital and classroom, the transmission risk shows higher than $10^{-3}$ in many cases, and a 2-m-distancing rule may not be effective to mitigate transmission, however, less stringent distancing is likely to be adequate in coach station and airport.

The work's limitations should be noted. The primary limitation is that only the airborne transmission risk of a susceptible receiver at short-range distance via inhalation of virus-laden respiratory droplets or droplet nuclei was evaluated. The risk of infection transmitted by spray and deposition of virus-laden large droplets, which is particularly significant at a very close distance (<0.5 m) is not included. Also, the pulsating exhalation flow is simplified to be steady in the present model, which may produce slightly modified results. The ambient air is assumed to be stagnant for predicting short-range transmission, and the factors affecting the long-range transmission such as the indoor airflow and ventilation rate (Li et al. 2021) are not involved in the present risk prediction model. Though numerous studies have reported that the existence of the air temperature gradient may extends the range of short-range airborne transmission, more detailed verifications of the close relevance between thermal stratification and short-range airborne transmission in real-world outbreak cases are needed in further studies. In addition, not all exposure scenarios were involved in the study; only some most commonly-seen cases were chosen for the risk assessment. The risk levels of SARS-CoV-2 in Figure 6 are only as an indication of relative risk, they could change especially in relation to the individuals' susceptibility to infection, shedding level from an infected person, and the personal hygiene habits and protections in public place. Further work is required to extend the risk stratify guide to develop specific policies to different types of indoor environments with various user behaviors.

**Electronic Supplementary Material:** the Appendix is available in the online version of this article at https://doi.org/10.1007/s12273-023-1021-5.

## Acknowledgements

This work was supported by Shanghai Sailing Program (No. 23YF1428700) and Sixue Program of University of Shanghai for Science and Technology (No. 2022-SX-036).

## Declaration of competing interest

The authors have no competing interests to declare that are relevant to the content of this article.

## Author contribution statement

All authors contributed to the study conception and design. Material preparation, data collection and analysis were performed by Fan Liu and Zhiwen Luo. The first draft of the manuscript was written by Fan Liu and all authors commented on previous versions of the manuscript. All authors read and approved the final manuscript.

**Open Access**: This article is licensed under a Creative Commons Attribution 4.0 International License, which permits use, sharing, adaptation, distribution and reproduction in any medium or format, as long as you give appropriate credit to the original author(s) and the source, provide a link to the Creative Commons licence, and indicate if changes were made.

The images or other third party material in this article are included in the article's Creative Commons licence, unless indicated otherwise in a credit line to the material. If material is not included in the article's Creative Commons licence and your intended use is not permitted by statutory regulation or exceeds the permitted use, you will need to obtain permission directly from the copyright holder.

To view a copy of this licence, visit http://creativecommons.org/licenses/by/4.0/

## Electronic supplementary material

Impact of thermal stratification on airborne transmission risk of SARS-CoV-2 in various indoor environments
