# Supplementary material for: Impact of thermal stratification on airborne transmission risk of SARS-CoV-2 in various indoor environments
Source: Build Simul. 2023 May 9:1–14. Online ahead of print. doi: 10.1007/s12273-023-1021-5 (PMC10166632; doi:10.1007/s12273-023-1021-5)
Supplement: Supplementary file 1 — Impact of thermal stratification on airborne transmission risk of SARS-CoV-2 in various indoor environments [file 12273_2023_1021_MOESM1_ESM.pdf]

Electronic Supplementary Material

Impact of thermal stratification on airborne transmission risk of SARS-CoV-2 in various indoor environments

Supporting information to https://doi.org/10.1007/s12273-023-1021-5

S1 Data collection of the vertical temperature from references

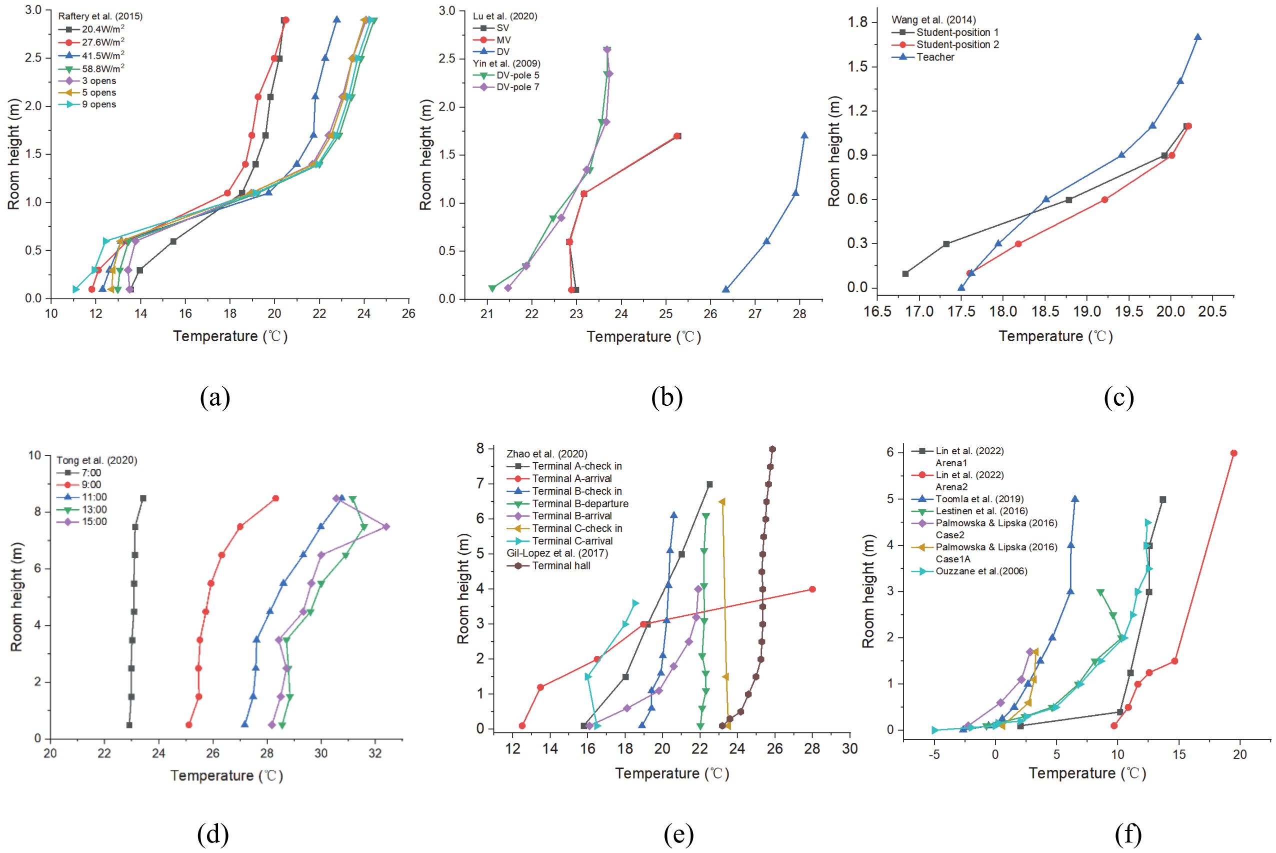

Fig. S1 Vertical temperature profiles retrieved from the literature in: (a) office (Raftery et al. 2015); (b) hospital (Yin et al. 2009; Lu et al. 2020); (c) classroom (Wang et al. 2014); (d) coach station (Tong et al. 2020); (e) airport (Gil-Lopez et al. 2017; Zhao et al. 2020); and (f) indoor ice rink (Ouzzane et al. 2006; Lestinen et al. 2016; Palmowska and Lipska 2016; Toomla et al. 2019; Lin et al. 2022)

S2 Risk assessment in different thermally stratified building environments

Table S1 Cross-infection risk of SARS-CoV-2 from asymptomatic people in different types of indoor environments

Building type	Exposure Scenario	Physical separation				
		0–0.5 m	0.5–1 m	1–1.5 m	1.5–2 m	> 2 m
**Office, short duration of exposure**						
Meeting room	Seated to seated	0.4099	0.192363333	0.058785	0.008446667	0.012698976
	Seated to standing	0	1.04961E−10	6.32327E−05	0.02107	0.044479474
**Office, long duration of exposure**						
Meeting room	Seated to seated	0.642975	0.347423333	0.114725	0.017173333	0.026064737
	Seated to standing	0	2.10275E−10	0.000126541	0.041606667	0.086598421
**Hospital**						
Consultation room	Seated to seated	0.047815	0.01918	0.00536	0.00067974	0.000581829
Dentist's office	Reclining to standing	1.02984E−14	9.33781E−07	3.28344E−07	1.14472E−06	4.94989E−07
Ward	Seated to seated	0.11379	0.045953333	0.01277	0.001521064	0.001430318
	Seated to standing	0	2.38534E−11	1.673E−05	0.00603872	0.010805263
	Lying to Lying	4.4186E−06	1.13842E−11	2.4721E−10	2.99958E−09	5.94935E−08
**Classroom**						
In class	Seated to standing	7.68935E−15	1.95793E−07	2.19039E−07	1.81117E−07	1.14167E−07
Break time	Seated side by side	0.08718	0.035313333	0.010405	0.001671459	0.003520056
**Bus station**						
Waiting hall	Seated to seated	3.69286E−07	7.32937E−13	1.24107E−14	1.78564E−12	2.01322E−11
	Seated to standing	7.9E−14	7.45033E−08	8.67239E−14	2.60238E−09	5.91472E−10
	Standing to standing	3.67677E−07	7.32833E−13	1.23251E−14	1.77333E−12	2.00509E−11
**Airport**						
Departure hall	Seated to seated	1.32463E−06	3.15574E−12	3.82895E−11	5.69103E−10	1.04893E−08
	Seated to standing	1.00304E−13	3.049E−07	2.94283E−08	2.94706E−07	1.15213E−07
	Standing to standing	1.32463E−06	3.37682E−12	3.82434E−11	5.69103E−10	1.04893E−08
Check-in hall	Standing to standing	4.41012E−08	1.71736E−13	1.39393E−12	1.8773E−11	3.48074E−10
**Ice rink**						
Competition field	Seated to seated	0.15842	0.024576667	4.96002E−05	4.56982E−09	0.000488671
	Seated to standing	0	2.21891E−05	0.02194	0.011802639	0.006045997

References

Gil-Lopez T, Galvez-Huerta MA, O’Donohoe PG, et al. (2017). Analysis of the influence of the return position in the vertical temperature gradient in displacement ventilation systems for large halls. *Energy and Buildings*, 140: 371–379.

Lestinen S, Koskela H, Jokisalo J, et al. (2016). The use of displacement and zoning ventilation in a multipurpose arena.

International Journal of Ventilation, 15: 151–166.

Lin W, Liu X, Zhang T, et al. (2022). Investigation of displacement and jet ventilation systems applied in an ice rink. *Journal of Building Engineering*, 50: 104179.

Lu Y, Oladokun M, Lin Z (2020). Reducing the exposure risk in hospital wards by applying stratum ventilation system. *Building and Environment*, 183: 107204.

Ouzzane M, Zmeureanu R, Scott J, et al. (2006). Cooling load and environmental measurements in a Canadian indoor ice rink. *ASHRAE Transactions*, 112(2): 538–545.

Palmowska A, Lipska B (2016). Experimental study and numerical prediction of thermal and humidity conditions in the ventilated ice rink arena. *Building and Environment*, 108: 171–182.

Raftery P, Bauman F, Schiavon S, et al. (2015). Laboratory testing of a displacement ventilation diffuser for underfloor air distribution systems. *Energy and Buildings*, 108: 82–91.

Tong Y, Lin K, Hu Q, et al. (2020). Field measurements on thermal stratification and cooling potential of natural ventilation for large space buildings. *International Journal of Ventilation*, 19: 49–62.

Toomla S, Lestinen S, Kilpeläinen S, et al. (2019). Experimental investigation of air distribution and ventilation efficiency in an ice rink arena. *International Journal of Ventilation*, 18:187–203.

Wang Y, Zhao F, Kuckelkorn J, et al. (2014). Classroom energy efficiency and air environment with displacement natural ventilation in a passive public school building. *Energy and Buildings*, 70: 258–270.

Yin Y, Xu W, Gupta J, et al. (2009). Experimental study on displacement and mixing ventilation systems for a patient ward. *HVAC & R Research*, 15: 1175-1191.

Zhao K, Weng J, Ge J (2020). On-site measured indoor thermal environment in large spaces of airports during winter. *Building and Environment*, 167: 106463.
